# Supplementary material for: Extended-Spectrum ß-Lactamase-Producing Escherichia coli in Conventional and Organic Pig Fattening Farms
Source: Microorganisms. 2022 Mar 11;10(3):603. doi: 10.3390/microorganisms10030603 (PMC8950372; doi:10.3390/microorganisms10030603)
Supplement: Supplementary file 1 [file microorganisms-10-00603-s001.zip › questionnaire.pdf]

Questionnaire

General information about the farm

1. production type and how many of them

- □ Sucking piglets
- □ Weaned piglets
- □ Rearing Pigs
- □ Gilts
- □ Sows
- □ Boars
- □ Fattening pigs

Fattening area

1. How many barn buildings are there?

2. when was the stable building built?

3. how many intended animal places are there in total in the barn under investigation?

4. how many intended animal places are there in total in the compartment?

5. how many age groups are there in these stalls?

6. how many pens are there in the compartment?

7. how many animals are currently in the compartment?

8. date of housing (of sampled animals)?

9. Type of husbandry
   - □ Conventional
   - □ Do you participate in any special programs?
     - □ Yes: ....
     - □ No
   - □ Organic
     - a) When did you switch to organic farming?
     - b) Do you belong to an association?
     - □ Yes:
     - □ No
10. What breed/s of pigs do you keep?
    ....

11. Which and how many of the listed animal species are still kept on this farm? Do these animals have contact with the pigs (□ yes/ □ no)?

□ Cattle
□ Poultry
□ Sheep
□ Goats
□ Horses
□ Dogs
□ Cats
□ Rabbits, guinea pigs
□ Others: ....

12. Which persons take care of the animals? (with number of employees, if applicable)

□ Farm manager
□ Family members
□ Trainees
□ Semi-skilled workers
□ Unskilled workers
□ Skilled workers
□ Others: ......

(a) Employees:

□ are responsible for only one particular barn
□ change between the stables
□ have access to all buildings of the farm.

(b) Is there a change of personnel on weekends, holidays or at vacation time?

□ Yes
□ No

13. Which persons have access to the inventory?

□ Farm employees
□ Veterinarian
□ Family members
□ Visitors (e.g., transporters, consultants, feeders, pest control, craftsmen).
□ Other individuals: ....

14. Do caregivers have private contact with other animals?

□ Yes, to: ....
□ No

<u>Specific information about the farm</u>

1. **How is the farm managed?**

□ closed system
□ piglet rearing operation with attached fattening operation
□ pure fattening operation

2. **Where do the fattening pigs come from?**

□ No animal purchases in the fattening area.
□ Purchases from one origin

- □ Purchases from several origins
- □ in one delivery
- □ in different deliveries
- □ from the region
- □ from further away
- □ always the same supplier
- □ different suppliers
- □ supplier last changed (date):
- □ Do you mix animals from different origins? (or do they stand separately in the barn)
  - □ Yes
  - □ No

3. **If closed system, how is the flat deck area usually occupied?**
   - □ Continuous
   - □ In-out
     - □ Pen
     - □ Stable

□ no spatial separation between flat-deck piglets and fattening pigs.

4. **how is the fattening area usually occupied?**
   - □ continuously
   - □ in-out
     - □ Pen
     - □ Stable
5. **how long is the fattening period?**

6. **Is there any rehousing during the fattening period?**
   - □ **Y**es
   - □ No
7. **Are individual pigs moved during the fattening period?**
   - □ **Y**es
   - □ No

<u>Surrounding area</u>

1. are there in the immediate vicinity (radius up to approx. 3 km)
   - □ Pig farms
   - □ Cattle farms
   - □ Poultry farms
   - □ Sheep and goat farms
   - □ Keeping horses
   - □ Water bodies with waterfowl
   - □ Wildlife and zoo animal holdings
   - □ Slaughterhouses
   - □ Processing plants
   - □ Sewage treatment plants

□ Rendering plants
□ Composting plants
□ Biogas plants
□ Marketing halls
□ Agricultural land
□ Forest
□ Hospital

Hygiene and cleaning

1. which hygiene measures are carried out before entering the stable?
   □ None
   □ Changing clothes/protective clothing/work clothes
   □ Gloves
   □ Hand cleaning
   □ Hand disinfection
   □ Boot cleaning
   □ Boot disinfection
   □ Boots are used ONLY for this barn and therefore are not cleaned every time.
2. how is access to the stalls done?
   □ There is no hygiene lock.
   □ There are disinfection mats/disinfection tubs for all stalls.
   □ Each stall has its own disinfection mat/disinfection tray.
3. what other protective measures do you have?
   □ None
   □ Fencing around the facility.
   □ Lockable doors
   □ Other: ......
4. is there a disinfection drive-through tub for vehicles?
   □ Yes
   □ No
5. which of the following items do you use for multiple stalls/exits without prior disinfection?
   □ Scales
   □ Feed truck
   □ Cleaning equipment
   □ Drift boards
   □ Sling
   □ Spray guns
   □ Marking pens
   □ Tools
   □ Ear marking pliers
   □ Other:....
   □ Does not apply
   □ Each barn has its own material.
6. do you share e.g. vehicles, machines or other equipment with other farms (joint use)?

□ Yes
□ No

7. When is cleaning and disinfection of compartments/outlets performed?

□ After each stabling
□ Less frequently than after each stall out.
□ Periodically, interval: ....
□ Never

8. When does cleaning and disinfection of drift paths outside of compartments occur?

□ Daily
□ Weekly
□ Monthly
□ After each stabling
□ Rarely
□ Never

9. Which includes cleaning and disinfecting the compartments?

□ Floor
□ Box partitions.
□ Walls at animal height
□ Walls up to the ceiling
□ Ceiling
□ Ventilation shafts
□ Windows
□ Outlet
□ Pasture
□ Piping system for drinking water
□ Watering troughs
□ Feed troughs/automatic feeders

10. What do you use to clean the stalls?

□ Pitchfork, broom, or similar.
□ Water hose
□ High-pressure cleaner
□ Other: ....

11. How long do you leave the cleaning agent to work?

□ < 1h
□ 1-4h
□ > 4h
□ Different
□ No cleaning agent is used.

12. Do you allow the surfaces to dry after cleaning?

□ Yes
□ No

13. How long do you leave the disinfectant on?

□ < 1h
□ 1-4h

□ > 4h
□ Different
□ No disinfectant is used.

14. What disinfectants do you use?

□ Acids
□ Aldehydes
□ Oxygen separators
□ Alcohols
□ Chlorine and chlorine releasers
□ Alkalis
□ Phenol derivatives
□ Quaternary ammonium compounds
□ Agents approved for organic farming
□ No disinfectant is used.
□ Others: .....

15. Is there a disinfection plan?

□ Yes
□ No

16. Which pests do you control and with what? 1=traps, 2= poison, 3= biological

□ Rodent pests, storage: ....
□ Flies
□ Other

17. Where is the manure/slurry stored?

□ Inside the building
□ Outside on the premises
□ Field
□ On concreted slab
□ In a closed container
□ In an open container
□ Unpaved ground in the yard.

18. Where are dead animals stored?

□ In the stall compartment
□ On the grounds outside the stall compartment.

<u>Diseases, use of drugs and antibiotics</u>

1. Can sick animals be housed in isolation from the others?
   □ Yes
   □ No
2. Where do sick pigs stay?
   □ In the pen
   □ they still have access to the run/pasture
   □ in a sick pen
   □ in a sick compartment, -stable
3. Is there a quarantine area?

□ Yes
□ No

4. Do you use homeopathics on your pigs and if so, how often?
    □ No
    □ Yes, rarely
    □ Yes, regularly
    □ Yes, very often
    □ Yes, always
5. Are your pigs treated with antiparasitics (e.g., Panacur, Rintal, Flubenol, Dectomax, Ivomec, Belamisol)?
    □ No
    □ No, but exercise and/or pasture areas are changed regularly.
    □ Yes, regularly
    □ Yes, irregularly
6. Please estimate how often the following diseases occur in your herd.
    a. Suckling piglets, weaners, finishing pigs, sows (never, rarely, often, regularly, always):
        □ Inflammation of the joints
        □ Hoof diseases
        □ Neurological diseases (fever, disturbed general condition, swelling of joints, animal holds head crooked, convulsions, animal lies sideways with rowing legs on the floor)
        □ Diarrhea
        □ Diseases of the respiratory system
        □ Skin diseases
    b. Sows additionally:
        □ Diseases of the urinary and genital system.
        □ Postpartum diseases (decrease or cessation of milk production, disturbed general condition, fever, inflammation of the mammary gland)
    c. General (never, rarely, often, regularly, always):
        □ Injuries to teats
        □ tail biting
        □ Cannibalism
        □ technopathies
7. Please estimate how often antibiotics are used
    □ Never
    □ Maximum once for fattening pigs
    □ Rarely
    □ Sometimes
    □ Often
    □ Always
8. Please estimate how antibiotics are used
    □ Group treatment (□ Never, □ Rarely, □ Sometimes, □ Often, □ Always).
    □ single animal treatment (□ never, □ rarely, □ sometimes, □ often, □ always).

9. Please estimate how the animals receive antibiotics
    □ Through feed (□ never, □ rarely, □ sometimes, □ often, □ always).

□ About the drinking water (□ never, □ rarely, □ sometimes, □ often, □ always).
Are the drinking water systems cleaned afterwards? □ Yes □ No
□ Via an injection (syringe).

10. What preparations/active ingredients are used?

11. Are your pigs vaccinated against?
    - □ Mycoplasma
    - □ Circoviruses (PCV2)
    - □ PRRSV
    - □ Lawsonia (ileitis)
    - □ Haemophilus parasuis (Glässers disease)
    - □ Actinobacillus pleuropneumoniae
    - □ vaccinated with herd-specific vaccines.
    - □ vaccinated with autovaccines.
12. Are your pigs treated with antibiotics before purchasing new animals?
    - □ Yes, always
    - □ No, never
    - □ Sometimes (how often, criteria?)
13. Are your animals treated with antibiotics before weaning?
    - □ Yes
    - □ No
    - □ Sometimes (how often, criteria?)
14. Are your animals treated with antibiotics before regrouping?
    - □ Yes
    - □ No
    - □ Sometimes (how often, criteria?).
15. Are your animals treated with antibiotics before regrouping?
    - □ Yes
    - □ No
    - □ Sometimes (how often, criteria?)
16. Are your animals treated with antibiotics before transport?
    - □ Yes
    - □ No
    - □ Sometimes (how often, criteria?)
17. If one animal in the group becomes ill and is given antibiotics, are the other animals in the group also treated with antibiotics as a precaution?
    - □ Yes
    - □ No
    - □ Sometimes (how often, criteria?).
18. Has the group of animals under study been treated with antibiotics since being housed?
    - □ Yes, all
    - □ Yes, individual animals (how often, criteria?)

      If yes, which antibiotic (animals examined/others)?

→ Diagnosis (A=respiratory, B=gastrointestinal, C=joints, D= CNS, E=skin, F=combat, G=claws, H=urinary and sexual tract, I=tail biting, cannibalism)?
→ How many animals?
→ How many days?
→ Which antibiotic?
→ Dosage per animal?
→ How administered (O=oral, S=syringe, L=local)?

□ No

19. Have the other groups of animals received antibiotic treatment since being housed?
    □ Yes, all
    □ Yes, individual animals
    □ No

20. What diseases are present in the herd at the time of the examination?

Husbandry

1. With what is the lying surface bedded?
   □ Cellulose products
   □ Spelt husks
   □ Straw
   □ Other: ......
   □ there is no interspersed lying area.
2. Is there a slatted floor?

   □ Yes
   □ No
3. Do your animals have a free range or live outdoors?

   □ Yes:
   □ → in summer
   □→ in spring
   □ → in autumn
   □→ in winter
   □ Year-round outdoor
   □ Free range
   □ No, neither outdoor nor free-range.
4. If outdoor or free-range:
   a. Does the run have a roof?

      □ yes, completely
      □ yes, partially
      □ no

   b. Is the floor in the run paved?

      □ Yes, completely

□ Yes, partially
□ No

c. What is the type of floor in the run or outdoor enclosure?

□ Pasture land
□ Earth soil
□ Concrete
□ Plastic
□ Metal
□ Is there additional burrowing material/litter (e.g., in the form of straw)?
□ Yes
□ No

d. Is the exercise area used by different animal groups (sows, fattening pigs, other animals) at the same time?

□ Yes
□ No

e. Is the exercise area used by different groups of animals consecutively?

□ Yes
□ No

f. Can mice, rats, and birds get into the exercise areas?

□ Yes: □ Mice □ Rats □ Birds
□ No

g. Where does the drinking water in the outdoor facility come from?

□ Public water supply
□ Well with drinking water quality
□ Well without testing (livestock well).
□ No extra water

h. Do livestock otherwise have access to water (e.g., stream/pond)?

□ Yes
□ No

i. Are there puddles or boggy areas in the run?

□ Yes, always
□ Yes, at times
□ No, never

5. What type of stall do you have?

□ Deep litter barns
□ Sloping floor barns
□ Open-front barns
□ Other: ....

6. Soil
   a. What is the type of floor in the barn?

- □ Fully slatted floor
- □ Partially slatted floor
- □ Full slatted floor with reduced slat percentage
- □ Floor fixed flat
- □ Deep litter/kicking manure method
- □ Other: ...

b. What material is the floor in the barn made of?

- □ Earthen floor
- □ Concrete
- □ Plastic
- □ Metal

7. What kind of ventilation is available?

   □ active (forced ventilation):
   □ passive (free ventilation):

8. What heating system is present?

   Heating type:

9. Temperature in the barn?

Feeding

1. Origin of the feed

   □ 100% company-owned
   □ Partly own
   □ purchased feed:
      □ 1 supplier
      □ several suppliers
         number of suppliers, constant or changing:

2. Do you use the farm's own basic feed (e.g. corn silage or green fodder) as supplementary feed?

   □ Yes
   □ No

3. Do you offer roughage, e.g. in the form of hay, green fodder or silage?

   □ Yes, everything from your own farm
   □ Yes, in proportions from your own farm
      number of suppliers, constant or changing?
   □ Yes, but not from own company
      number of suppliers, constant or changing?
   □ No

4. Feeding technique

   □ Rationed
   □ Ad libitum
   □ Trough feeding by hand
   □ Brewers

□ Liquid feeding
□ Sensor feeding
□ Automatic dry feeder
□ Other: ...

5. How and where is the feed stored?

- ...

Water supply

6. Origin of the drinking water
   □ Public water supply
   □ Well with drinking water quality
   □ Wells without testing
7. What types of watering troughs are used?
   □ Nipple drinkers
   □ Cup drinkers/bowl drinkers
   □ Trough

Crop farming

1. do you practice crop farming?
   □ Yes
   □ No
2. do you apply liquid manure/slurry/manure to the arable land?
   □ Yes: - only farm-owned
   □ on-farm and off-farm
   □ Off-farm only
   □ No
3. Do you apply liquid manure/slurry/manure to the grassland areas?
   □ Yes
   □ No
4. Do you grow cereals?
   □ Yes: exclusively from own farm.
   If yes, is straw from fields fertilized with liquid manure/slurry/manure used in your own livestock production?
   □ From own farm as well as bought in
   □ No
5. Is feed produced from these fertilized fields on your own farm and fed to your animals?
   □ Yes
   □ No
6. Manure removal
   □ Liquid manure
   □ Solid manure

Biogas plant

1. Is this one present?
   □ Yes
   □ No
2. Which substrate is used?
   □ Liquid manure
   □ Manure
   □ ....
3. Is only the farm's own material used?
   □ Yes
   □ No
4. Supplier farms:
   Animal species: ....
   Farm type: ....
   Production type (e.g. organic): ....
5. Is the digestate applied to your own cropland/grassland?
   □ Yes
   □ No

Performance parameters

Daily gain:

Feed conversion ratio:

Loss rate:

Fattening runs per year:
