# Supplementary material for: Extended-Spectrum ß-Lactamase-Producing Escherichia coli in Conventional and Organic Pig Fattening Farms
Source: Microorganisms. 2022 Mar 11;10(3):603. doi: 10.3390/microorganisms10030603 (PMC8950372; doi:10.3390/microorganisms10030603)
Supplement: Supplementary file 1 [file microorganisms-10-00603-s001.zip › questionnaire_results.pdf]

	Farm 1	Farm 2	Farm 3	Farm 4a	Farm 4b	Farm 5	Farm 6	Farm 7
**Production type**	fattening	closed system	Piglet rearing and fattening	fattening	fattening	fattening	Piglet rearing and fattening	closed system
**Number of fattening pigs**	3,500	4,600	2,600	500	1,000	1,280	1,000	1,400
**Number of barn buildings**	2	4	4	3	4	2	4	1
**Year of construction**	2002, 2006	late 1980ies<br>1 Building 2010	1970ies	1970ies	1970ies	2008/2009, 2018	1970ies, 1998	1975
**Stocking frequency**	continuously	continuously	Irregular (depending on capacity)	Irregular (depending on availability of suppliers)	Irregular (depending on availability of suppliers)	Irregular (depending on availability of suppliers)	Irregular (depending on availability of suppliers)	continuously
**Within a radius of up to 3km**<br>**- Pig farms**<br>**- Water bodies with waterfowl**	 +<br>+	 -<br>+	 -<br>+	 +<br>+	 +<br>+	 -<br>-	 -<br>+	 -<br>+
**Care of the pigs**	Farm Manager<br>Family members<br>Unskilled workers	Farm Manager<br>Family members<br>Trainees<br>Semi-skilled workers	Farm Manager<br>Skilled staff	Skilled staff	Skilled staff	Skilled staff	Farm Manager<br>Family members<br>Trainees<br>Unskilled workers	Farm Manager<br>Specialized staff<br>Semi-skilled workers
**Responsibility of employees**	Access to all buildings of the farm	Rotation between the stables	Access to all buildings of the farm	Rotation between the stables	Rotation between the stables	Access to all buildings of the farm	Access to all buildings of the farm	Only for specific stable
**Change of personnel on weekends, holidays, in vacation time**	+	–	+	+	+	+	-	-
**Access for visitors (e.g. transporters, feed consultants)**	+	+<br>School classes	+	-	-	+	+<br>School classes<br>Kindergartens	+
**Private pig farming of employees**	Forbidden	Forbidden	Forbidden	Forbidden	Forbidden	Forbidden	Not forbidden	Forbidden
**Participation in quality programs**	QS-System	Tierwohl	QS-System	Biopark	Biopark	Biokreis	Biopark	Naturland

Purchase of fattening pigs	1 origin	No purchase	4 origins	2 origins	1 origin	2 origins	1 origin	No purchase
**Duration of fattening period in weeks**	16-18	13	16	18-19	18-19	14-17	17	14-20
**Cleaning and disinfection of the compartments and outlets after each fattening run.**	+	+	+	-	-	+	+	-
**Cleaning and disinfection of driveways**	As needed	After each fattening run	As needed	No driveways	No driveways	No driveways	Weekly	After each fattening run
**hygiene sluice for each stable**	+	+	+	-	-	+	-	-
**disinfection mats for each stable**	- (stable specific boots)	- (stable specific boots)	+	+	+	+	+	- (stable specific boots)
**Use of homeopathic drugs**	-	-	-	-	-	-	+	-
**Use of antiparasitic drugs**	-	+ (irregular)	+ (irregular)	-	-	+ (regular)	+ (regular)	-
**Vaccination**	+ PCV2, *Mycoplasma*	+ PCV2, *Haemophilus parasuis*	+ PCV2, *Mycoplasma* PRRSV	+ *Erysipelothrix rhusiopathiae*	+ *Erysipelothrix rhusiopathiae*	+ *Erysipelothrix rhusiopathiae*	+ PCV2, *Mycoplasma, Lawsonia,* STEC	+ PCV2, *Mycoplasma*, STEC, *Salmonella* Typhimurium
**Use of antibiotic drugs**	+	+	+	+	+	+	+	+
**Frequency of the use of antibiotic drugs (estimate of the farm manager)**	rarely	rarely	often	rarely	sometimes	rarely	sometimes	rarely
**Group treatment with antibiotic drugs**	-	-	+	-	+	-	+	-

**Application route of antibiotic drugs**	Injection	Injection	Injection (individual animals) via feed/drinking water (group)	Injection	Injection	Injection	Injection (individual animals) Via drinking water (piglets)	Injection
**Antibiotic substances used**	Tylosin Cefquinom	Amoxicillin Sulfonamid Ceftiofur Tulathromycin Colistin	Fattening pigs: Chlortetracycline Sulfamethoxazole Marbofloxacin Cefquinom Piglets: Amoxicillin Doxycyclinhyclat Lincomycin/Spectinomycin Marbfloxacin Enrofloxacin Tylosin	Tylosin Tulathromycin	Ceftiofur Tulathromycin	Procain-Benzyl-penicillin Enrofloxacin Sulfadimidin/ Trimethoprim	Tiamulin Tylosin Ceftiofur Enrofloxacin Colistinsulfat (Ferkel)	Amoxicillin Tulathromycin Marbofloxacin
